# Inflammatory bowel disease therapies and demyelinating diseases: a practical guide to therapeutic benefit and risk

**DOI:** 10.1093/ecco-jcc/jjaf215

**Published:** 2025-11-29

**Authors:** Sailish Honap, Marc Debouverie, Massimo Filippi, Daniel Selchen, Vipul Jairath, Silvio Danese, Laurent Peyrin-Biroulet

**Affiliations:** Department of Gastroenterology, St George’s University Hospitals NHS Foundation Trust, London, United Kingdom; School of Immunology and Microbial Sciences, King’s College London, London, United Kingdom; Department of Neurology, Nancy University Hospital, Nancy, F-54 000, France; Neurology Unit, Multiple Sclerosis Centre, IRCCS San Raffaele Scientific Institute, Milan, 20132, Italy; Neurorehabilitation Unit, IRCCS San Raffaele Scientific Institute, Milan, Italy; Vita-Salute San Raffaele University, Milan, Italy; Neuroimaging Research Unit, Division of Neuroscience, IRCCS San Raffaele Scientific Institute, Milan, Italy; Division of Neurology, Department of Medicine, St Michael’s Hospital, Toronto, Ontario, Canada; Division of Gastroenterology, Department of Medicine, Western University, London, Ontario, Canada; Department of Epidemiology and Biostatistics, Western University, London, Ontario, Canada; Department of Gastroenterology and Digestive Endoscopy, IRCCS Ospedale San Raffaele, Milan, Italy; Department of Gastroenterology, CHRU Nancy, INSERM NGERE, Université de Lorraine, Vandœuvre-lès-Nancy, F-54500, France

**Keywords:** inflammatory bowel disease, demyelinating disease, multiple sclerosis, monoclonal antibodies

## Abstract

Demyelinating diseases, particularly multiple sclerosis (MS), present a unique therapeutic challenge in the management of inflammatory bowel disease (IBD). Although rare, the co-occurrence of IBD and demyelinating disorders is well-documented and may reflect shared immune, genetic, and environmental risk factors. As the therapeutic landscape of IBD expands to include biologics and small molecules that target immune pathways also implicated in MS, concerns around neurological safety have grown. In particular, anti-tumor necrosis factor agents have been consistently linked to new-onset or worsening demyelinating events, while other treatments such as sphingosine-1-phosphate receptor modulators and natali­zumab are licensed for both IBD and MS, though real-world data in patients with coexisting disease remain limited. This review synthesizes current evidence regarding the neurological safety and efficacy of IBD therapies in the context of demyelinating disease. It proposes a practical framework for clinicians, addressing management strategies for patients with confirmed MS, those at increased risk, and individuals who develop neurological symptoms during treatment. In the absence of formal guidelines, multidisciplinary collaboration, early recognition of symptoms, and careful treatment selection are important to optimize both gastrointestinal and neurological outcomes.

## 1. Introduction

Inflammatory bowel diseases (IBD), primarily ulcerative colitis (UC) and Crohn’s disease (CD), are chronic, progressive, and potentially disabling immune-mediated inflammatory diseases (IMIDs) of the gastrointestinal tract.^1^^,^^2^ Up to one in four individuals with IBD also have a concurrent IMID, which may precede or follow the onset of gastrointestinal symptoms.^3^ Demyelinating diseases are a heterogeneous group of neurological disorders affecting the central and peripheral nervous systems, characterized by the damage of the myelin sheath that insulates nerve fibers. These include multiple sclerosis (MS)—the most prevalent and a leading cause of non-traumatic disability in young adults^4^—and also conditions such as optic neuritis and transverse myelitis, which may present in isolation or as early manifestations of a chronic demyelinating disorder. An association between IBD and demyelinating diseases, particularly MS, has been hypothesized for several decades.^5^

The global incidence and prevalence of both IBD and MS are increasing, underscoring a growing burden of IMIDs across disciplines.^5^^,^^6^ Epidemiological studies suggest that individuals with IBD have a 4-fold increased risk of developing demyelinating disorders, particularly MS, compared to the general population.^7^ The prevalence of MS in patients with IBD is estimated at 0.2%, while the prevalence of IBD in patients with MS is higher at approximately 0.6%.^8^ Although the epidemiological association between IBD and MS is well established,^9^ the underlying mechanisms remain poorly defined. The coexistence of MS and IBD is thought to reflect shared autoimmune mechanisms, along with overlapping genetic and environmental risk factors. However, the incomplete understanding of these pathways complicates clinical management, as treatments effective for one condition may inadvertently worsen the other.

In addition to corticosteroids and 5-aminosalicylates, the expanding therapeutic landscape of IBD includes biologics and small molecules that modulate immune pathways also implicated in demyelinating disease, including TNF, JAK-STAT, and IL-12/23 signaling.^10^^,^^11^ Anti-tumor necrosis-alpha (TNF-α) agents, in particular, have been associated with the unmasking or worsening of demyelination in some patients.^12^ Meanwhile, therapies such as sphingosine-1-phosphate (S1P) receptor modulators—licensed in both gastroenterology and neurology–further complicate treatment decisions in those with coexisting disease. As such, IBD physicians face uncertainty when managing patients with confirmed or suspected demyelinating conditions, as well as those at increased risk, including individuals with a strong family history of MS.

Given the absence of formal guidelines or expert consensus, this review aims to clarify the occurrence and clinical impact of demyelinating disorders associated with therapies used in IBD, identify potential therapeutic overlaps, and support safe, personalized treatment strategies. While several reviews have examined the use of MS therapies in the treatment of IBD and other autoimmune conditions, and these are briefly discussed for completeness, no review to date has focused specifically on the impact and management of IBD therapies in patients with MS.^13–15^ Hence, a comprehensive literature search was conducted across MEDLINE, EMBASE, CENTRAL, and ClinicalTrials.gov from inception to May 2025 to identify studies examining the association between therapies used in IBD and demyelinating disorders. Eligible studies reported potential therapeutic benefit, adverse neurological outcomes, or insufficient evidence to determine the neurological impact of IBD therapies in demyelinating conditions. Full eligibility criteria and search strategies are provided in the Supplementary Material.

## 2. Drugs with therapeutic benefit across both IBD and demyelinating diseases

### 2.1. Corticosteroids

Corticosteroids are central to managing acute flares in both IBD and demyelinating diseases. In IBD, they are used in the short term for induction, while in MS and related disorders, high-dose corticosteroids are standard for treating relapses.^16^ They are not linked to demyelination and are considered neurologically safe. In patients with coexisting disease or when therapeutic decisions are complex, corticosteroids can serve as a bridging option while longer-term treatments are considered.

### 2.2. Sphingosine-1-receptor modulators

S1P is a bioactive lipid derived from sphingolipid metabolism at the cell membrane, acting through a family of five G protein-coupled receptors (S1P1-S1P5).^17^ S1P signaling pathways play a critical and multifaceted role in homeostasis and are key mediators of lymphocyte trafficking, cardiovascular and neurological function, vascular permeability, and bronchial tone.^18^ They have emerged as therapeutic targets across a range of IMIDs due to their role in regulating immune cell trafficking, particularly by controlling the egress of lymphocytes from lymph nodes into the circulation. Although S1P receptor modulators are an emerging treatment class in IBD, they are well established for the management of MS.

Among S1P receptor modulators approved for the treatment of relapsing MS, fingolimod was the first to be approved for clinical use, demonstrating a significant reduction in relapse rates and magnetic resonance imaging (MRI) lesion activity.^19^ However, its broad receptor affinity targeting S1P1, S1P3, S1P4, and S1P5 was associated with off-target effects such as bradycardia and cardiac conduction abnormalities.^19^ This led to the development of second-generation S1P receptor modulators with greater receptor selectivity and improved safety profiles, such as ponesimod and ozanimod. Ozanimod, which selectively targets S1P1 and S1P5, was designed to maintain immunomodulatory efficacy while reducing cardiovascular risks. The efficacy of ozanimod in MS was demonstrated in two pivotal phase 3 trials, which showed significant reductions in annualized relapse rates and MRI lesion activity compared with interferon beta-1a.^20^^,^^21^ These findings confirmed the therapeutic utility of selective S1P1 and S1P5 modulation in relapsing disease, leading to regulatory approval. Ozanimod is indicated for the treatment of clinically isolated syndrome, relapsing–remitting, and active secondary progressive MS. For UC, the efficacy of ozanimod was established in the phase 3 True North trial, which evaluated its use in patients with moderate-to-severe disease.^22^ In both induction and maintenance phases, ozanimod demonstrated significant improvements compared with placebo in clinical, endoscopic, and histologic endpoints.^22^ The drug was generally well tolerated, with most adverse events being mild to moderate and consistent with the known class effects, including transient bradycardia and elevated liver enzymes. These findings supported its regulatory approval for UC in 2021.^23^

Ozanimod is the only currently approved therapy with regulatory indications for both MS and IBD. However, evidence supporting its use in patients with coexisting IBD and demyelinating disorders remains scarce and is restricted to individual case reports.^24^^,^^25^ Etrasimod is another selective S1P receptor modulator targeting S1P1, S1P4, and S1P5 with efficacy in UC and was approved in 2024 following the ELEVATE UC 52 and ELEVATE UC 12 trials.^26^ Despite its shared mechanism of action, etrasimod has not been studied in MS or other demyelinating diseases, and no studies were identified that have assessed its neurological safety or efficacy in these conditions.

Beyond ozanimod and etrasimod, other S1P receptor modulators such as amiselimod and tamuzimod are being explored for the treatment of IBD.^27^^,^^28^ However, no eligible studies or ongoing trials were identified evaluating these agents in demyelinating disorders. The approval of ozanimod for both IBD and MS highlights a potential therapeutic option for patients with coexisting disease, highlighting the need for dedicated studies in this population.

### 2.3. Anti-leukocyte trafficking agents

Anti-leukocyte trafficking agents act by blocking integrins involved in lymphocyte adhesion and migration across endothelial barriers, thereby limiting immune cell infiltration into target tissues.^29^ This mechanism underlies their use in IBD and demyelinating disorders, where aberrant leukocyte trafficking contributes to chronic inflammation. Among the three anti-integrin therapies currently licensed for IBD^30^—natalizumab, vedolizumab, and carotegrast methyl—only natalizumab is also approved for use in MS, with regulatory approval granted by the U.S. Food and Drug Administration (FDA) but not the European Medicines Agency (EMA). Natalizumab is a humanized monoclonal antibody that targets the α4 subunit of integrins, blocking both α4β1–VCAM-1 and α4β7–MAdCAM-1 interactions. By inhibiting these adhesion pathways, it prevents lymphocyte trafficking across the endothelium into the central nervous system (CNS) and gastrointestinal tract. In contrast, vedolizumab selectively inhibits the α4β7–MAdCAM-1 interaction, restricting its activity to the intestinal mucosa, while carotegrast methyl (AJM300) is an oral, reversible α4-integrin antagonist that transiently blocks VCAM-1 and MAdCAM-1 binding, producing a functionally gut-selective effect with minimal systemic immune suppression.

The search identified four phase III randomized controlled trials (RCTs) evaluating natalizumab for the treatment of relapsing–remitting or active secondary progressive MS, all of which demonstrated efficacy in reducing clinical relapses and MRI disease activity.^31–34^ Multiple observational studies identified have since reinforced its effectiveness in real-world settings.^35^^,^^36^ Natalizumab is also licensed for use in CD in the USA following trials that demonstrated efficacy in inducing and maintaining clinical response in moderate-to-severe CD.^37^^,^^38^ However, its uptake in clinical practice has been limited due to the associated risk of progressive multifocal leukoencephalopathy.^39^ Risk mitigation strategies, including routine screening for JC virus seropositivity, are essential when considering natalizumab in clinical practice.

Vedolizumab, with its gut-selective mechanism and favorable safety profile, may be used in combination with approved MS therapies for patients requiring treatment of both IBD and demyelinating disease, with the exception of natalizumab due to overlapping mechanisms of action. Evidence directly supporting this approach, however, remains limited, and data on the combined or parallel use of approved therapies for MS and IBD are scarce overall and are isolated to case reports.^40^^,^^41^

## 3. Therapies with possible therapeutic overlap or uncertain evidence base

### 3.1. Thiopurines

Thiopurines (azathioprine and 6-mercaptopurine) are immunosuppressive agents widely used in the management of IBD and other IMIDs, particularly for steroid-sparing maintenance in moderate-to-severe disease. They exert their immunomodulatory effects primarily by inhibiting purine synthesis and lymphocyte proliferation.

A total of eight randomized controlled trials were identified evaluating the efficacy of azathioprine in MS.^42–49^ Across all trials, azathioprine consistently demonstrated modest efficacy in reducing relapse frequency in MS. Most studies reported a 20%-40% reduction in relapse rates compared with placebo or interferon comparators, with several trials reaching statistical significance. However, improvements in disability progression were generally limited or inconsistent, and treatment was sometimes associated with increased adverse events (Table 1). Overall, azathioprine provides measurable though moderate benefit in relapse prevention, supporting its efficacy while highlighting its relatively modest impact on long-term disability outcomes. This, alongside availability of disease-modifying therapies with superior efficacy and safety profiles, has largely limited the use of thiopurines in the treatment of MS.^4^

**Table 1.** Summary of published phase II/III randomized controlled trials of approved IBD therapies in demyelinating diseases.

Therapy	Lead author (year)	*N*	Design	Intervention	Disease	Primary endpoint	Key findings
**Azathioprine**	British and Dutch MS Trial Group (1988)^42^	354	Double blind, randomized, PBO controlled	AZA 2.5 mg/kg vs PBO	RRMS	KDSS and ambulation index at three years	Minimal benefit. AZA reduced relapse rates by 21% over 3 years (*P* = .03) compared to placebo in patients with MS. No significant difference in progression of EDSS or Ambulation Index, indicating limited impact on disability.
	Kappos (1988)^47^	194	Double blind, randomized, controlled	AZA 2.5 mg/kg vs CsA 5 mg/kg	RRMS	Relapse rate and KDSS	Minimal benefit. Reduced MS relapse rate from 1.23 to 0.98 per year (*P = *.03) but had no significant impact on disability progression (EDSS change: AZA 0.36 vs placebo 0.42).
	Ellison (1989)^43^	98	Double blind, randomized, PBO controlled	AZA + PBO vs AZA + MP	PPMS	Mickey’s Illness Severity Scores and KDSS at 3 years	Minimal benefit but AEs. AZA + MP reduced MS relapse rates by up to 44% over 3 years (*P = *.03) but offered minimal benefit on disability and was associated with increased AE.
	Goodkin (1991)^45^	59	Double blind, PBO controlled	AZA 3 mg/kg vs PBO	RRMS	Mean exacerbation rate after 2 years and time to deterioration in Ambulation Index and KDSS	AZA significantly reduced exacerbation rates in year 2 (mean 0.30 vs 0.79, *P* = .05) and prolonged time to deterioration in EDSS and Ambulation Index compared to placebo (*P* = .04 and *P* = .03 respectively).
	Milanese (1992)^49^	40	Double blind, randomized, PBO controlled	AZA 2 mg/kg	RRMS, PPMS, SPMS	Relapse-free survival, and disability progression from baseline using EDSS at 3 years	AZA reduced EDSS worsening (38% vs 82% deteriorated; *P* = .051) and increased relapse-free patients (40% vs 10%; *P* = .07), though differences did not reach statistical significance.
	Etemadifar (2007)^44^	94	Single blind, randomized, controlled	AZA 3 mg/kg vs IFNβ (variable dosing based on product used)	RRMS	Relapse-free survival, and disability progression from baseline using EDSS at 1 year	AZA reduced mean annual relapse rate (0.28 vs 0.64, *P <*.05) and improved EDSS more than interferon beta (−0.46 vs −0.30), with 77% vs 57% of patients remaining relapse-free (*P <* .05).
	Havrdova (2009)^46^	181	Double blind, randomized, PBO controlled	AZA 2.5 mg/kg vs IFNβ 250 μg every other day	RRMS	Time to first relapse over the 24-month treatment period	AZA was inferior to interferon beta-1b, with 56% of azathioprine-treated patients experiencing relapse vs 37% on interferon (*P = *.03), and disability progression in 39% vs 16% respectively (*P = *.01).
	Massacesi (2014)^48^	127	Single blind, randomized, controlled	AZA 2.5 mg/kg vs IFNβ 250 μg every other day	RRMS	Annualized relapse rate ratio over 2 years	Annualized relapse rate was 0.26 (95% CI, 0.19-0.37) in the AZA and 0.39 (95% CI 0.30-0.51) in the IFN group. Non-inferiority analysis showed that AZA was at least as effective as interferon (relapse RRAZA/IFN 0.67, one-sided 95% CI 0.96; *P* < .01).
**Methotrexate**	Currier (1993)^59^	44	Double blind, randomized, PBO controlled	MTX 7.5 mg/week vs PBO	RRMS, SPMS	The number and timing of exacerbations and worsening of at least one point on the Kurtzke Disability Scale over the treatment period	In patients with RRMS, MTX reduced the mean number of exacerbations from 12 in 11 placebo patients to 3 in 9 MTX patients, with a significant reduction in combined exacerbations and ≥1-point Kurtzke scale worsening (*P* = .05), while no benefit was observed in SPMS patients.
	Goodkin (1995)^58^	60	Double blind, randomized, PBO controlled	MTX 7.5 mg/week vs PBO	PPMS, SPMS	Composite outcome of: EDSS, ambulation index, Box and Block Test, and 9-Hole Peg Test.	Low-dose MTX significantly reduced sustained treatment failure over 2 years compared to placebo (51.6% vs 82.8%; *P* = .011).
	Ashtari (2011)^60^	80	Single blind, randomized, controlled	MTX 7.5 mg/week vs IFN-β-1α 30 μg/week	RRMS	The change in relapse rate over 12 months of treatment	MTX significantly reduced relapse rate from 1.75 to 0.97 over 12 months in RRMS patients, but was less effective than interferon-beta-1α, which reduced relapse rate from 1.52 to 0.57 (*P* = .06 for between-group difference).
**Sulfasalazine**	Noseworthy (1998)^106^	199	Double blind, randomized, PBO controlled	Sulfasalazine 3 g/day vs PBO	RRMS, PPMS, SPMS	Worsening of EDSS score by at least 1 point on two consecutive 3-month visits	Sulfasalazine did not significantly reduce relapse rate (0.96 vs 1.01 relapses/year for placebo; *P = *.87) or disability progression over 2 years in active MS patients.
**Natalizumab**	Miller (2003)^31^	213	Double blind, randomized, PBO controlled	3–6 mg/kg NTZ vs PBO	RRMS, SPMS	Number of new brain lesions on monthly gadolinium-enhanced MRI over 6 months	Marked reductions in mean number of new lesions in both NTZ groups: 9.6 per patient in the PBO group vs. 0.7 in the 3 mg NTZ group (*P* <.001) and 1.1 in the group given 6 mg NTZ/kg (*P* <.001).
	O’Connor (2004)^32^	180	Double blind, randomized, PBO controlled	Single dose of NTZ at 1 or 3 mg/kg or PBO	RRMS	Change in neurological disability, as measured by the EDSS at week 14	NTZ significantly improved EDSS scores at 14 weeks compared to PBO (mean improvement 1.6 vs 0.9; *P = *.004) and reduced the number of new MRI lesions in acute MS relapses.
	Polman (2006)^33^	627	Double blind, randomized, PBO controlled	NTZ q4 weekly vs PBO	RRMS	Rate of clinical relapse at 1 year and disability progression as per EDSS	NTZ significantly reduced relapse rate by 68% and risk of disability progression by 42% vs to PBO at 1 year.
	Rudick (2006)^34^	1171	Double blind, randomized, PBO controlled	NTZ 300 mg q4 weekly + IFN-β vs. PBO + IFN-β	RRMS	Rate of clinical relapse and EDSS at 2 years	NTZ added to IFN-β was significantly more effective than IFN-β alone. Combination therapy reduced relapse rates by 24% and risk of disability progression by 28% compared to IFN-β alone.
**Ustekinumab**	Segal (2008)^102^	249	Double blind, randomized, PBO controlled	Four doses (27 mg, 90 mg q8w, 90 mg, or 180 mg) given at weeks 0, 1, 2, 3, 7, 11, 15, and 19.	RRMS	Number of new active MRI T2-weighted lesions at week 12.	Ustekinumab is generally well tolerated but did not demonstrate efficacy in reducing MRI T2-weighted lesion activity in patients with relapsing–remitting MS.
**Ozanimod**	Comi (2019)^20^	1346	Double blind, randomized, active-controlled	Ozanimod 0.5 or 1 mg vs IFN-β 30 µg	RRMS	Annualized relapse rate	Ozanimod significantly reduced annualized relapse rate compared to interferon beta-1a (0.18 vs 0.35; rate ratio 0.53, 95% CI 0.41-0.69; *P* <.0001) over a median 13.8-month follow-up in relapsing MS patients.
	Cohen (2019)^21^	1320	Double blind, randomized, active-controlled	Ozanimod 1 mg vs IFN-β 30 µg	RRMS	Annualized relapse rate	Ozanimod significantly reduced the annualized relapse rate compared to interferon beta-1a over 24 months in relapsing MS patients (0.22 vs 0.40; rate ratio 0.57; 95% CI 0.44-0.74; *P* <.0001)

Abbreviations: AE, adverse events; AZA, azathioprine; CI, confidence interval; CsA, ciclosporin A; EDSS, Expanded Disability Status Scale; IFNβ, interferon beta; KDSS, Kurtzke Expanded Disability Status Scale score; MP, methylprednisolone; MRI, magnetic resonance imaging; MTX, methotrexate; MS, multiple sclerosis; NTZ, natalizumab; PBO, placebo; PPMS, primary progressive multiple sclerosis; RRMS, relapsing remitting multiple sclerosis; SPMS, secondary progressive multiple sclerosis.

Azathioprine has also been shown to be effective in the treatment of neuromyelitis optica spectrum disorder (NMOSD), an autoimmune condition of the CNS that can present with optic neuritis and transverse myelitis. Its use has been associated with reduced relapse rates and disability progression, as demonstrated in an RCT and several prospective studies.^50–54^ Similarly, benefit has also been observed in chronic inflammatory demyelinating polyradiculoneuropathy (CIDP), although the evidence base is limited.^55^^,^^56^ Therefore, thiopurines may remain a reasonable option in select patients with demyelinating diseases, particularly those with comorbid IBD or other overlapping considerations in jurisdictions where better treatments are not available or affordable.

### 3.2. Methotrexate

Methotrexate (MTX) is a folate antagonist with anti-proliferative and immunosuppressive properties, commonly used in the treatment of autoimmune diseases. In IBD, MTX is used primarily as a concomitant immunomodulator and while there is some evidence supporting its use in CD, targeted therapies with superior efficacy and safety profiles are preferred.^57^ Three RCTs across relapsing–remitting or progressive MS have previously demonstrated either a significant benefit or a positive, albeit non-significant, trend toward reduced relapse rates and disability, supporting its potential role in disease management.^58–60^ However, as in IBD, the emergence of more potent and targeted therapies has limited its clinical use. For other demyelinating disorders, such as CIDP, one RCT showed significant benefit though limitations in the trial design and the high rate of response in the placebo group meant that a treatment effect could not be excluded.^61^ In NMOSD, retrospective observational studies have reported that MTX may reduce relapse frequency and stabilize disability progression, although controlled data are lacking.^62^^,^^63^

Taken together, MTX is unlikely to offer substantial therapeutic benefit across demyelinating diseases but appears to be neurologically safe as it is not associated with neurotoxicity or the induction of demyelination.

### 3.3. Calcineurin inhibitors

Calcineurin inhibitors, including cyclosporine and tacrolimus, are potent immunosuppressants that inhibit T-cell activation by blocking the calcineurin pathway. In IBD, cyclosporine is typically used for steroid-refractory acute severe UC, while tacrolimus has historically been utilized in chronic, active UC, often in patients who are refractory to conventional therapies.^64^ Calcineurin inhibitors have also been used in demyelinating diseases, though their role is very limited. In MS, older studies evaluated cyclosporine but efficacy was modest and toxicity and safety concerns limited long-term use.^65^^,^^66^ Tacrolimus has been used anecdotally in NMOSD and other neuroinflammatory conditions, often in the context of transplantation or refractory cases.^67–69^

Calcineurin inhibitors have not been associated with inducing demyelinating disease and are generally considered safe in this context. In patients with severe UC and coexisting demyelinating conditions, calcineurin inhibitors may be an appropriate short-term bridging strategy, particularly where anti-TNF-α agents are contraindicated.

## 4. Therapies associated with neurological harm or lack of benefit

While some IBD therapies may offer overlapping benefit or at least neurological safety in patients with coexisting demyelinating disorders, others have been linked to potential neurological harm or have shown no evidence of benefit in this context. The following section examines such therapies, with an emphasis on reported associations with demyelination.

### 4.1. Anti-TNF agents

Since their introduction in the mid- to late 1990s, anti-TNF-α agents have remained among the most widely used immunosuppressive therapies in IBD and across the spectrum of IMIDs, owing to their broad anti-inflammatory effects and well-established clinical efficacy. However, soon after their widespread adoption, numerous reports began to emerge of new-onset neurologic signs and symptoms associated with demyelinating events affecting the central and peripheral nervous systems. While such events are relatively rare, their potential severity and occasionally irreversible outcomes have prompted concern regarding a possible causal relationship. In the current era of multiple biologic and small molecule options, this concern is less of a restrictive dilemma than previously—yet it continues to influence clinical decision-making in patients with suspected or established MS, particularly when anti-TNFs might otherwise be appropriate.

Early studies suggested that elevated levels of TNF-α played a pathological role in the development and progression of MS. Increased concentrations of TNF-α were detected in the cerebrospinal fluid of patients with MS, correlating with disease activity and neurological decline.^12^ These observations led to the hypothesis that TNF-α was a key driver of CNS inflammation and tissue damage, making it an attractive therapeutic target in early experimental approaches. However, in one of the earliest case series, two patients with aggressive, intravenous steroid-refractory MS who received infliximab, then referred to as cA2, demonstrated an unexpected increase in the number of gadolinium-enhancing lesions on MRI following each infusion, compared to baseline.^70^ A subsequent RCT of lenercept, a soluble TNF receptor fusion protein, in patients with relapsing–remitting MS was terminated prematurely due to a significant increase in clinical relapses and MRI activity in the treatment group compared to placebo.^71^ Mechanistically, this paradox may be explained, in part, by the dual role of TNF-α in neuroinflammation. While soluble TNF signals through TNFR1 to promote inflammation and oligodendrocyte death, membrane-bound TNF-α activates TNFR2, which is essential for oligodendrocyte progenitor cell survival, differentiation, and remyelination within the CNS.^72^^,^^73^ Another proposed mechanism suggests that, because TNF-α inhibitors have limited penetration across the blood–brain barrier, they selectively suppress TNF activity in peripheral tissues while leaving central TNF signaling relatively unopposed.^12^^,^^74^ This compartmentalized inhibition may paradoxically result in increased TNF activity within the CNS.

Accumulating real-world evidence over the past two decades has continued to support a potential association between anti-TNF-α therapy and demyelinating events. Numerous case reports and case series have described new-onset central and peripheral nervous system demyelination, including MS, optic neuritis, transverse myelitis, Guillain-Barré syndrome, and CIDP, occurring in temporal association with all anti-TNF-α agents licensed for IBD.^75–91^ Demyelination associated with TNF-α blockers does not appear to correlate reliably with treatment duration, and clinical improvement is not consistently observed following drug discontinuation.^92^ Population-based cohort studies show individuals with IBD have a 2- to 4-fold increased risk of developing TNF-α-associated demyelinating conditions, though the absolute risk is low.^7^^,^^93^^,^^90^ Importantly, 9%-17% of patients who developed TNF-α-related demyelination had a near or distant family history of MS.^85^^,^^94^

Further insight into the clinical phenotype of anti-TNF-α-associated demyelination was provided by a multicenter case-control study of 48 affected patients and 1219 anti-TNF-α-exposed controls. Most affected individuals were female and presented with CNS demyelination after a median of 21 months of therapy.^94^ Most patients (55%) required treatment for demyelination, most commonly corticosteroids, but also intravenous immunoglobulin and plasma exchange. Complete recovery was reported in 23% of cases, after a median follow-up time of 6.8 months, and partial recovery occurred in 55% of patients after a median follow-up of 33 months. However, a subset experienced persistent or progressive neurologic impairment with up to 3 years of post-treatment follow up. Genetic analysis revealed no significant difference in MS susceptibility scores between cases and controls, suggesting that these events probably arise from treatment-related mechanisms rather than underlying genetic risk.^94^ Notwithstanding these observations, it remains unclear whether TNF-α blockers unmask pre-existing MS, provoke *de novo* demyelinating disease, or whether these observations are a result of a greater predisposition or developing another IMID. Histopathologically, anti-TNF-α-induced inflammatory demyelinating activity has been shown to be indistinguishable from MS.^95^

Given these concerns, anti-TNF-α agents are generally avoided in patients with a personal history of demyelinating disease and used with caution in those with suggestive symptoms or a strong family history. Alternative monoclonal antibodies or small molecules with no known neurological risk may be preferred. When anti-TNFs are used, clinicians should maintain a high index of suspicion for neurologic symptoms and consider prompt referral for neuroimaging and specialist evaluation if concerns arise.

### 4.2. Anti-interleukin 12/23 agents

Ustekinumab is a fully human monoclonal antibody targeting the p40 subunit shared by interleukin (IL)-12 and IL-23. It is approved for the treatment of moderate to severe CD and UC with a well-established safety profile supported by both clinical trials and real-world evidence.^96–98^

Early studies showed that IL-12 and IL-23 were strongly implicated in the pathogenesis of MS.^99–101^ A subsequent double‑blind, placebo‑controlled phase II RCT in patients with relapsing–remitting MS found that ustekinumab did not reduce the cumulative number of gadolinium-enhancing T1 lesions on MRI compared to placebo by week 23.^102^ The treatment was well tolerated, with similar adverse event rates to placebo and no exacerbation of demyelinating disease.^102^ However, despite these findings, three isolated case reports have described new-onset demyelination temporally associated with ustekinumab exposure, suggesting that rare idiosyncratic events may still occur.^103–105^ These reports underscore the importance of ongoing pharmacovigilance, although they do not appear to alter the overall favorable neurological safety profile demonstrated in controlled trials and large post-marketing cohorts.

### 4.3. 5-Aminosalicylates

5-Aminosalicylates (5-ASAs), such as mesalazine and sulfasalazine, are widely used in mild-to-moderate UC but have no therapeutic role in demyelinating diseases. They are considered neurologically safe and are not associated with central demyelination. The only randomized trial of sulfasalazine in MS—a double-blind study by Noseworthy et al.—found no clinical or radiological benefit compared to placebo, demonstrating the lack of efficacy in this setting.^106^ As such, 5-ASAs are viewed as safe but ineffective in the context of demyelinating disease.

## 5. Agents without evidence regarding benefit or risk in demyelinating diseases

### 5.1. Janus kinase inhibitors

Janus kinase (JAK) inhibitors are a class of small-molecule therapies that modulate intracellular signaling by blocking one or more members of the JAK family, JAK1, JAK2, JAK3, and TYK2.^107^ JAKs play a central role in transducing signals from a variety of pro-inflammatory cytokines involved in autoimmune and inflammatory processes. In IBD, tofacitinib and the more selective upadacitinib, and filgotinib have demonstrated efficacy and are now approved for use in moderate to severe disease.^107^

Given their mechanism of action and growing use across multiple IMIDs, there has been interest in their potential application to neurological diseases, including MS. Studies in murine models of experimental autoimmune encephalomyelitis (EAE) have been mixed. JAK inhibition has been shown to markedly reduce inflammatory demyelination by limiting granulocyte–macrophage colony-stimulating factor-mediated recruitment of inflammatory monocytes and monocyte-derived dendritic cells into the CNS, thereby impairing their capacity for antigen presentation.^108^ A separate pre-clinical study showed that baricitinib significantly delayed the onset time, decreased the severity of clinical symptoms, shortened the duration of EAE, and alleviated demyelination and immune cell infiltration in the spinal cord.^109^ However, low doses of tofacitinib have also accelerated EAE through the activation of Th17 cells and excessive production of IL-17.^110^

Clinical data on the neurological safety of JAK inhibitors remain limited, and there are no published or ongoing trials investigating their safety or efficacy in demyelinating diseases. Nevertheless, three isolated case reports have described the emergence of demyelinating syndromes temporally associated with tofacitinib therapy, raising the possibility of a rare but clinically relevant adverse effect in susceptible individuals.^111–113^

### 5.2. Anti-interleukin 23 agents

Anti-IL-23 (p19) monoclonal antibodies are increasingly used in the management of IBD. No eligible studies were identified evaluating their utility in MS or their potential association with demyelinating disease. No actively recruiting trials evaluating this drug class were identified on ClinicalTrials.gov. Table 1 summarizes the published trials of IBD therapies in demyelinating diseases, while Table 2 summarizes the efficacy and safety ratings of IBD therapies in demyelinating diseases.

**Table 2.** Efficacy and safety rating for approved IBD therapies in the context of demyelinating diseases.

Therapy class	Drug	First approved in IBD	Efficacy and safety rating
**Corticosteroid**	Prednisolone	1955	++
**Corticosteroid**	Methylprednisolone	1957	++
**Thiopurine**	Azathioprine	1968	+
**Antimetabolite**	Methotrexate	1989	±
**Aminosalicylate**	Mesalazine	1987	Insufficient evidence
**Aminosalicylate**	Sulfasalazine	1950s	±
**Anti-TNF**	Infliximab	1998 (CD) 2005 (UC)	—
**Anti-TNF**	Adalimumab	2007 (CD) 2012 (UC)	—
**Anti-TNF**	Certolizumab pegol	2008 (UC)	—
**Anti-TNF**	Golimumab	2013 (UC)	—
**Anti-Integrin**	Natalizumab	2008 (CD)	++
**Anti-Integrin**	Vedolizumab	2014 (CD and UC)	Insufficient evidence
**Anti-IL-12/23**	Ustekinumab	2016 (CD) 2019 (UC)	−
**S1PR modulator**	Ozanimod	2021 (UC)	++
**S1PR modulator**	Etrasimod	2023 (UC)	Insufficient evidence
**JAK inhibitor**	Tofacitinib	2018 (UC)	–
**JAK inhibitor**	Filgotinib	2021 (UC)	Insufficient evidence
**JAK inhibitor**	Upadacitinib	2023 (CD) 2022 (UC)	Insufficient evidence
**Anti-IL-23**	Risankizumab	2022 (CD) 2024 (UC)	Insufficient evidence
**Anti-IL-23**	Mirikizumab	2025 (CD) 2023 (UC)	Insufficient evidence
**Anti-IL-23**	Guselkumab	2025 (CD and UC)	Insufficient evidence

++ Strong evidence of neurological safety or benefit in demyelinating disease.

+ Moderate or suggestive evidence of safety or benefit.

± Neurologically neutral; no evidence of harm or benefit.

− Some concern or isolated reports of demyelination.

— Strong or consistent association with neurological harm.

Abbreviations: CD, Crohn’s disease; IL, interleukin; JAK, Janus kinase; S1PR, sphingosine-1-receptor; TNF, tumor necrosis factor; UC, ulcerative colitis.

## 6. Other disease-modifying agents in MS and their impact on IBD

**Figure 1.**
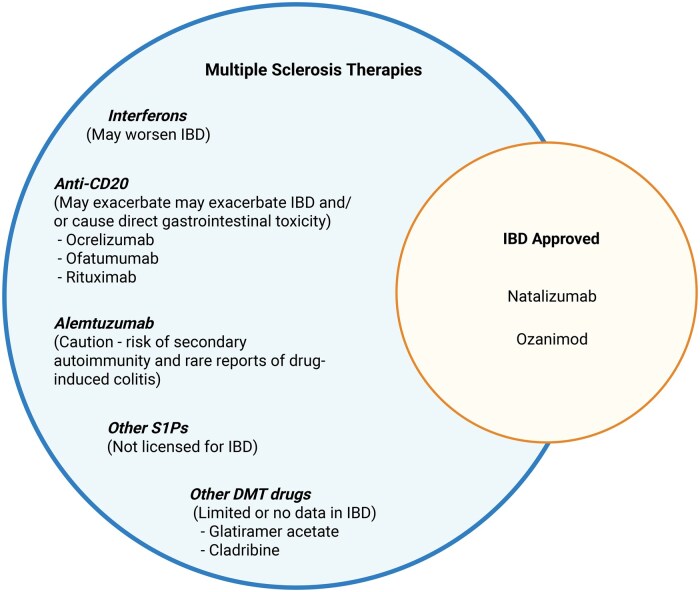
Disease-modifying therapies for multiple sclerosis and their implications in IBD. This schematic illustrates selected multiple sclerosis (MS) disease-modifying therapies (DMTs) in relation to inflammatory bowel disease (IBD) safety and efficacy. Therapies positioned in the blue circle are established MS treatments, with annotations indicating their reported effects in IBD. Only natalizumab and ozanimod (highlighted in orange) are approved for both MS and IBD, representing the limited therapeutic overlap. Other MS DMTs either lack efficacy in IBD or carry safety concerns, including worsening of gastrointestinal disease.

While this review focuses primarily on the neurological safety and efficacy of IBD therapies, this section briefly considers the reverse scenario—how established MS disease-modifying therapies, beyond S1P receptor modulators and natalizumab, may influence intestinal inflammation. These agents are briefly summarized here for context, as they have been reviewed in detail elsewhere (Figure 1).^13–15^

Interferons are a well-established and widely used therapy for MS and modulate immune activity by reducing pro-inflammatory cytokine production, limiting T-cell activation and migration across the blood–brain barrier.^114^ Clinical studies of interferon therapy in UC and CD have yielded inconsistent results, with early reports suggesting benefit but subsequent controlled trials showing no meaningful or sustained efficacy.^14^ Isolated case reports have also described new-onset or exacerbation of disease following interferon treatment.^115–117^

Monoclonal antibodies targeting CD20, including ocrelizumab and ofatumumab, achieve selective B-cell depletion and have become central to the management of MS.^118^ Experience with rituximab led to the development of these newer humanized antibodies with better tolerability and formal approval for use in MS.^118^ However, anti-CD20 therapies are generally not favored in IBD and may contribute to immune dysregulation and subsequent inflammation in the gut. An Icelandic population-based study reported a 6-fold increased risk of developing IBD with rituximab, and a previous RCT showed no significant effect on the proportion of patients who achieve remission in moderately active UC.^119^^,^^120^ Furthermore, several case reports have described *de novo* colitis and CD with ocrelizumab and ofatumumab, suggesting a possible class effect.^121–124^ These observations highlight that anti-CD20 therapies are probably best avoided in IBD and that clinicians should remain alert to the potential for *de novo* intestinal inflammation during treatment.

Alemtuzumab is a monoclonal antibody directed against CD52 that causes deep, sustained depletion of T and B lymphocytes, followed by gradual immune reconstitution that can dampen immune activity but may also trigger secondary autoimmune disease.^125^ Alemtuzumab carries a well-recognized risk of secondary autoimmunity, and rare cases of alemtuzumab-induced colitis have been reported, warranting clinical awareness of this potential complication.^126^^,^^127^

## 7. Summary and practical approach to demyelination risk in IBD therapy

**Figure 2.**
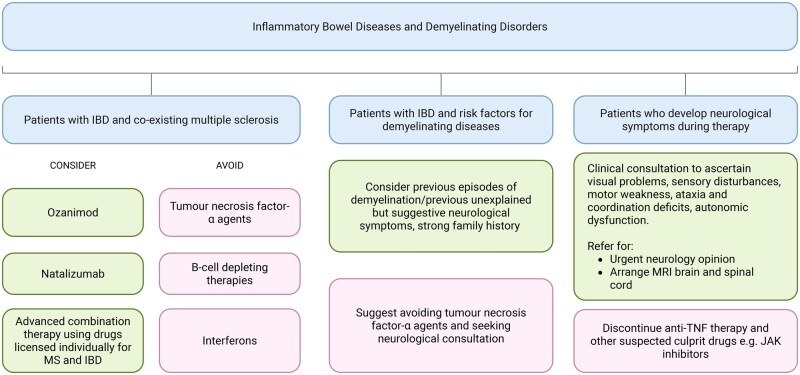
Pharmacological considerations for IBD and demyelinating diseases overlap. This schematic outlines a recommended strategy for managing patients who have both IBD and overlapping demyelinating diseases. For patients with coexisting inflammatory bowel disease (IBD) and multiple sclerosis (MS), only ozanimod and natalizumab are approved to treat MS and ulcerative colitis or Crohn’s disease, respectively. Drugs approved for each condition with favorable safety profiles may be combined, but evidence for this strategy is extremely limited. B-cell-depleting therapies and interferons are best avoided as they have been shown to cause direct gastrointestinal toxicity and exacerbate existing IBD and cause *de novo* presentations of IBD. Patients should be informed of the rare risk of demyelination with anti-TNF therapy and healthcare providers should be vigilant in those with pre-existing risk factors and in those who develop neurological symptoms during therapy.

Clinicians managing patients with IBD must occasionally navigate therapeutic decisions in the context of suspected or confirmed demyelinating disease. Compared to other IMIDs, demyelinating disorders such as MS share relatively little therapeutic overlap with IBD, creating a unique clinical dilemma when both conditions coexist. The expanding therapeutic landscape in IBD includes agents that interact with immune pathways shared by demyelinating diseases, creating potential for both therapeutic synergy and neurological risk. In the absence of formal guidelines, the evidence reviewed in this paper supports a practical framework to guide treatment decisions in these complex scenarios. This final section briefly summarizes the available evidence and offers practical guidance to support clinical decision-making where demyelination risk is a concern (Figure 2). Of note, only natalizumab and ozanimod are currently approved for both conditions, while other MS disease-modifying therapies either lack efficacy in IBD or carry important safety concerns.

### 7.1. Patients with confirmed demyelinating disease

In patients with a confirmed diagnosis of MS, therapeutic decision-making should prioritize agents with a well-established neurological safety profile. Anti-TNF-α therapies are generally contraindicated in this population due to consistent associations with new-onset or worsening demyelinating events. Even in the absence of active neurological symptoms, their use may pose an unacceptable risk of disease reactivation or progression.

In patients with UC and coexisting MS, ozanimod may be a reasonable option given its regulatory approval and demonstrated efficacy in both conditions; however, clinical evidence specifically addressing its use in individuals with overlapping disease remains limited. In patients with both CD and MS, where a monoclonal antibody is indicated for the management of both conditions, and in regions where natalizumab is approved for use in IBD, this agent may be considered as a single therapeutic option, particularly in JC virus-negative individuals. This approach may offer a streamlined strategy for dual disease control in selected cases.

All other treatment classes for IBD may be considered acceptable in patients with coexisting demyelinating disease, including conventional immunosuppressants. Therapeutic decisions should be guided by disease severity, phenotype, and prior treatment history. Drugs approved for each condition with favorable safety profiles may be combined, but evidence for this strategy remains extremely limited. Multidisciplinary collaboration with neurology is recommended, and close monitoring of both gastrointestinal and neurological disease activity is essential throughout therapy.

### 7.2. Patients with risk factors for demyelinating disease

Demyelinating events associated with IBD therapy are uncommon, with data from large registries and population-based cohorts suggesting an absolute risk of fewer than 1 per 1000 patients exposed to anti-TNF-α blockers.^86^ Engaging in shared decision-making is essential; patients should be informed of the potential, albeit rare, risk of neurological complications and involved in weighing this against the benefits of treatment.

In individuals with potential risk factors—such as unexplained neurological symptoms (past or present), a confirmed or suspected prior demyelinating event, or a strong family history of MS or related disorders—it may be prudent to adopt a more cautious approach. While a positive family history may raise clinical suspicion, it is not a reliable predictor of individual risk. Most individuals with a family history will not develop demyelinating disease, and many affected patients have no known genetic predisposition, reflecting the complex interplay of genetic, environmental, and epigenetic factors.^4^ Evaluating this risk is highly complex, with little data to inform risk–benefit discussions. Performing screening MRI on all such patients considering anti-TNF-α therapy is not feasible from a resource perspective, and the use of MRI in this way confers a risk of significant anxiety in patients as a result of incidental diagnoses with varying degrees of clinical significance being revealed.

In patients with active neurological symptoms, further investigation, including neurological assessment and MRI imaging, should be pursued prior to therapy initiation. A thorough history and neurology input may also be appropriate in those with historical or familial risk factors, particularly when considering agents temporally linked to demyelinating events, such as anti-TNF therapies. Although these factors do not preclude the use of effective IBD treatments, they do justify a more cautious, individualized approach supported by multidisciplinary collaboration.

### 7.3. Patients who develop neurological symptoms during therapy

The emergence of neurological symptoms during immunosuppressive treatment for IBD presents a diagnostic and therapeutic challenge. While demyelinating complications remain rare, clinicians should maintain a high index of suspicion for new-onset neurological symptoms, particularly in patients receiving anti-TNF-α agents. Typical presenting features of demyelination include optic neuritis (visual blurring, reduced visual acuity, and retro-orbital pain), motor weakness (asymmetric limb weakness or monoparesis), sensory disturbance (paresthesia, dysesthesia, or hypoesthesia), ataxia (gait unsteadiness or limb incoordination), and sphincter dysfunction (neurogenic bladder symptoms such as urgency, incontinence, or retention). Peripheral demyelination, as seen in CIDP, typically presents with progressive, symmetric limb weakness, areflexia, and distal sensory loss.

The aforementioned symptoms should prompt urgent neurological evaluation to exclude central or peripheral demyelinating disease. As an immediate step, the suspected culprit drug should be withheld. MRI of the brain and spinal cord with contrast is essential to characterize the pattern and extent of demyelination and to guide further management. At present, no evidence-based guidelines exist for the treatment of drug-associated demyelinating events. Acute treatment typically involves high-dose intravenous corticosteroids. In patients who demonstrate a relapsing–remitting course, further investigation for underlying autoimmune or primary demyelinating disorders is warranted, and long-term immunosuppressive therapy may be required. Rechallenge with the suspected agent is ill-advised, given the potential for recurrent or more severe neurological complications. Selection of an alternative IBD therapy should be guided by disease severity, prior treatment history, and the treatment required for demyelinating disease.

## 8. Conclusions

As therapeutic options for IBD continue to expand, clinicians should consider the neurological safety profiles of immunosuppressive agents, particularly in patients with confirmed or suspected demyelinating disorders. While most therapies appear to carry minimal risk, anti-TNF-α agents remain uniquely associated with new-onset or worsening demyelination and should generally be avoided in high-risk individuals. For patients with established MS, S1P receptor modulators and anti-integrins (natalizumab) are licensed for use in both IBD and MS. However, despite regulatory approval in both disease areas, clinical evidence supporting their effectiveness when treating both conditions concurrently remains limited. In the absence of formal guidelines, a pragmatic, case-by-case approach grounded in emerging evidence and interdisciplinary collaboration remains essential. This review highlights the importance of early recognition, risk stratification, and tailored therapeutic planning to optimize both gastrointestinal and neurological outcomes in complex clinical scenarios. Further research is needed to identify risk factors and better characterize individuals who may be susceptible to de novo demyelinating disease or exacerbation of pre-existing demyelinating pathways in the context of immunomodulatory therapy.

## Supplementary Material

jjaf215_Supplementary_Data

## Data Availability

No new data were generated for this review.
