# Supplementary material for: Inflammatory bowel disease therapies and demyelinating diseases: a practical guide to therapeutic benefit and risk
Source: J Crohns Colitis. 2025 Nov 29;20(1):jjaf215. doi: 10.1093/ecco-jcc/jjaf215 (PMC12803786; doi:10.1093/ecco-jcc/jjaf215)
Supplement: jjaf215_Supplementary_Data [file jjaf215_supplementary_data.docx]

**Supplementary Information**

# Methods

## Search Strategy and Eligibility Criteria

A scoping search was conducted to identify studies reporting the association between IBD therapies and demyelinating disorders, including both the risk of adverse neurological events and the potential therapeutic use of these agents in demyelinating conditions. The search was conducted in MEDLINE and EMBASE via the Ovid platform, the Cochrane Central Register of Controlled Trials (CENTRAL), and ClinicalTrials.gov from inception to 1 May 2025. The search strategies combined terms for IBD, demyelinating disorders, and relevant therapy classes. No language restrictions were applied. Details of full search strategies are provided below in the Supplementary Material. Studies were eligible for inclusion if they reported on the occurrence, risk, or potential benefit of demyelinating disorders in the context of exposure to treatments used for IBD. Eligible study designs included randomized controlled trials (RCTs), cohort studies, case-control studies, case series and case reports. Conference abstracts, narrative reviews, and editorials were excluded.

## Study Selection, Data Extraction and Data Synthesis

All identified records were reviewed to determine study eligibility based on the predefined inclusion and exclusion criteria. Extracted data reviewed included study design, population characteristics, type and duration of IBD therapy, nature of demyelinating events (e.g., diagnosis, timing, severity), therapeutic response (if applicable), and relevant confounders (e.g., history of demyelination, family history of MS). Given the anticipated heterogeneity in study populations, therapies, and outcome definitions, a descriptive synthesis was undertaken. Studies were grouped by IBD therapy class and demyelinating disorder subtype. Both adverse neurological outcomes and potential therapeutic effects on demyelinating disorders were summarized.

# Search Strategies

## MEDLINE and Embase Search Strategies

A comprehensive literature search was conducted to identify studies reporting the association between IBD therapies and demyelinating disorders, including both the risk of adverse neurological events and the potential therapeutic use of these agents in demyelinating conditions. MEDLINE and EMBASE databases were searched via the Ovid platform from inception to April 30, 2025.

The search strategy combined MeSH/Emtree terms and free-text keywords for:

- Inflammatory bowel disease (Crohn’s disease, ulcerative colitis, IBD-unclassified),
- IBD therapies (including anti-TNF agents, anti-integrins, interleukin-12/23 and anti-p19 agents, JAK inhibitors, S1P receptor modulators, and immunomodulators), and
- Demyelinating diseases (including multiple sclerosis, optic neuritis, transverse myelitis, ADEM, neuromyelitis optica, and MOG antibody-associated disease).

To account for differences in database structure and indexing, each search was tailored:

- In MEDLINE, a broader strategy that did not restrict the population to IBD was used, in order to capture rare safety signals or trials in other autoimmune populations potentially relevant to IBD practice.
- In EMBASE, which yielded a higher volume of off-target results and conference abstracts, the population was restricted to IBD. Free-text fields were limited to title and abstract (.ti,ab.) to reduce indexing noise.

Search strategies were iteratively tested against known key references. In addition to database searches, a hand-search of reference lists was performed from all included full-text articles and relevant review papers to identify additional eligible studies not captured by electronic searches. The full search strategies are provided below.

## MEDLINE Search Strategy

| 1. | Infliximab/ or Adalimumab/ or Certolizumab Pegol/ or Golimumab/ |
| --- | --- |
| 2. | (infliximab or adalimumab or certolizumab or golimumab or anti TNF or TNF inhibitor*).tw,kf. |
| 3. | 1 or 2 |
| 4. | Vedolizumab/ or Natalizumab/ |
| 5. | (vedolizumab or natalizumab or anti integrin* or integrin inhibitor*).tw,kf. |
| 6. | 4 or 5 |
| 7. | Ustekinumab/ |
| 8. | (ustekinumab or guselkumab or risankizumab or mirikizumab or anti IL-23 or anti p19).tw,kf. |
| 9. | 7 or 8 |
| 10. | JAK Inhibitors/ or Tofacitinib/ or Upadacitinib/ or Filgotinib/ |
| 11. | (JAK inhibitor* or tofacitinib or upadacitinib or filgotinib).tw,kf. |
| 12. | 10 or 11 |
| 13. | Sphingosine-1-Phosphate Receptor Modulators/ or Ozanimod/ |
| 14. | (S1P modulator* or ozanimod or etrasimod).tw,kf. |
| 15. | 13 or 14 |
| 16. | Azathioprine/ or Methotrexate/ or Mercaptopurine/ |
| 17. | (azathioprine or methotrexate or mercaptopurine).tw,kf. |
| 18. | 16 or 17 |
| 19. | 3 or 6 or 9 or 12 or 15 or 18 |
| 20. | Demyelinating Diseases/ or Multiple Sclerosis/ or Optic Neuritis/ or Transverse Myelitis/ or Acute Disseminated Encephalomyelitis/ or Neuromyelitis Optica/ or Myelin-Oligodendrocyte Glycoprotein/ |
| 21. | (demyelinating or demyelination or "multiple sclerosis" or MS or "optic neuritis" or "transverse myelitis" or "acute disseminated encephalomyelitis" or ADEM or "neuromyelitis optica" or NMO or Devic* or "MOG antibody disease" or MOGAD or "central demyelination" or "CNS demyelination" or "myelin oligodendrocyte").tw,kf. |
| 22. | 20 or 21 |
| 23. | 19 and 22 |
| 24. | 23 not (editorial or comment or letter).pt. |
| 25. | limit 24 to (humans and english language and yr="1995 -Current") |

## EMBASE Search Strategy

| 1. | infliximab/ or adalimumab/ or certolizumab pegol/ or golimumab/ |
| --- | --- |
| 2. | (infliximab or adalimumab or certolizumab or golimumab or anti TNF or TNF inhibitor*).ti,ab. |
| 3. | 1 or 2 |
| 4. | vedolizumab/ or natalizumab/ |
| 5. | (vedolizumab or natalizumab or anti integrin* or integrin inhibitor*).ti,ab. |
| 6. | 4 or 5 |
| 7. | ustekinumab/ |
| 8. | (ustekinumab or guselkumab or risankizumab or mirikizumab or anti IL-23 or anti p19).ti,ab. |
| 9. | 7 or 8 |
| 10. | jak inhibitor/ or tofacitinib/ or upadacitinib/ or filgotinib/ |
| 11. | (JAK inhibitor* or tofacitinib or upadacitinib or filgotinib).ti,ab. |
| 12. | 10 or 11 |
| 13. | sphingosine 1 phosphate receptor modulator/ or ozanimod/ |
| 14. | (S1P modulator* or ozanimod or etrasimod).ti,ab. |
| 15. | 13 or 14 |
| 16. | azathioprine/ or methotrexate/ or mercaptopurine/ |
| 17. | (azathioprine or methotrexate or mercaptopurine).ti,ab. |
| 18. | 16 or 17 |
| 19. | 3 or 6 or 9 or 12 or 15 or 18 |
| 20. | demyelinating disease/ or multiple sclerosis/ or optic neuritis/ or transverse myelitis/ or acute disseminated encephalomyelitis/ or neuromyelitis optica/ or myelin oligodendrocyte glycoprotein/ |
| 21. | (demyelinating or demyelination or "multiple sclerosis" or MS or "optic neuritis" or "transverse myelitis" or "acute disseminated encephalomyelitis" or ADEM or "neuromyelitis optica" or NMO or Devic* or "MOG antibody disease" or MOGAD or "central demyelination" or "CNS demyelination" or "myelin oligodendrocyte").ti,ab. |
| 22. | 20 or 21 |
| 23. | 19 and 22 |
| 24. | 23 not (editorial or letter or note or conference abstract or conference review).pt. |
| 25. | inflammatory bowel disease/ or crohn disease/ or ulcerative colitis/ or (IBD or "inflammatory bowel disease" or Crohn* or "ulcerative colitis").ti,ab. |
| 26. | 24 and 25 |
| 27. | limit 26 to (human and english language) |

## Cochrane CENTRAL Search Strategy

A search of the Cochrane Central Register of Controlled Trials (CENTRAL) was conducted in April 2025 via the Cochrane Library interface to identify randomized controlled trials evaluating therapies commonly used in IBD for the treatment or safety evaluation of demyelinating diseases. These included multiple sclerosis (MS), neuromyelitis optica (NMO), myelin oligodendrocyte glycoprotein antibody disease (MOGAD), acute disseminated encephalomyelitis (ADEM), optic neuritis, and transverse myelitis. Separate keyword searches were performed for each IBD-related drug, using the following structure:

Search format:

"Drug name" AND ("multiple sclerosis" OR "demyelinating disease" OR "neuromyelitis optica" OR "ADEM" OR "optic neuritis" OR "transverse myelitis" OR "MOGAD")

The therapies searched included:

- Anti-TNF agents: infliximab, adalimumab, certolizumab pegol, golimumab
- Anti-integrins: vedolizumab, natalizumab
- IL-12/23 and anti-p19 (IL-23) agents: ustekinumab, guselkumab, risankizumab, mirikizumab
- JAK inhibitors: tofacitinib, upadacitinib, filgotinib
- S1P modulators: ozanimod, etrasimod
- Immunomodulators: azathioprine, methotrexate, mercaptopurine

Each drug was searched in combination with the specified demyelinating disease terms. For example:

"ustekinumab" AND "multiple sclerosis"
"tofacitinib" AND ("demyelinating disease" OR "multiple sclerosis")

Results were manually screened to confirm relevance and trial design. Eligible studies were randomized controlled trials that evaluated the selected therapies in the context of central nervous system demyelinating disease. No filters were applied to limit by language or publication date. All trials labeled as randomized or indexed from recognized trial registries (e.g., ClinicalTrials.gov, WHO ICTRP) were included.

## Clinicaltrials.gov Search Strategy

A systematic search of ClinicalTrials.gov (accessed May 2025) was conducted to identify interventional studies evaluating therapies used in IBD in the context of demyelinating disorders. The following neurological conditions were used as search terms, individually and in combination: demyelinating diseases, multiple sclerosis, optic neuritis, transverse myelitis. Each condition term was paired with the name of an IBD-related therapy, including: azathioprine, methotrexate, 6-mercaptopurine, infliximab, adalimumab, certolizumab, golimumab, vedolizumab, natalizumab, ustekinumab, guselkumab, risankizumab, mirikizumab, tofacitinib, upadacitinib, filgotinib, ozanimod, and etrasimod. A filter was applied to restrict results to trials that were actively recruiting or seeking participants at the time of the search. Observational-only studies were excluded. Eligible trials included those evaluating one of the listed therapies with outcomes related to demyelinating disease.

In addition to database searches, we performed hand-searching of reference lists from all included full-text articles and relevant review papers to identify additional eligible studies not captured by electronic searches.
